# Synergetic inhibitory effect of isopropyl methylphenol-based agents on biofilm formation by *Streptococcus mutans*

**DOI:** 10.1371/journal.pone.0310926

**Published:** 2024-09-23

**Authors:** Arisu Korenaga, Toshiki Miyaoka, Harumi Asami, Yasushi Yamagami, Miki Yoshii, Shigemitsu Tanaka, Toshihiro Nagao

**Affiliations:** 1 Research & Development division, Osaka kasei Co., Ltd., Osaka, Japan; 2 Research Division of Biomaterials and Commodity Chemicals, Osaka Research Institute of Industrial Science and Technology, Osaka, Japan; University of Pennsylvania, UNITED STATES OF AMERICA

## Abstract

Dental caries and periodontitis are the most common oral diseases in humans and the main causes of tooth loss. *Streptococcus mutans* is primarily responsible for dental caries and dental plaque, which are triggered by biofilm formation on the tooth surface. In this study, biofilm inhibition by 4-isopropyl-3-methylphenol (IPMP)-based agents, consisting of IPMP and polyoxyethylene-hydrogenated castor oil (POEHCO), was investigated in vitro. Notably, the use of POEHCO in addition to IPMP inhibited *S*. *mutans* biofilms more drastically than IPMP alone. Moreover, the effects of IPMP on the expression of biofilm-related genes (*gtfB*, *gtfC*, and *gtfD*) were examined using quantitative real-time PCR. IPMP at sub-minimum inhibitory concentrations significantly downregulated the expression of these genes. These results suggested that the inhibitory effects on biofilm formation were synergistically enhanced by the surfactant and antibiofilm activities of IPMP. Therefore, IPMP-based agents as dentifrices may be useful to prevent oral diseases originating from biofilms.

## Introduction

Dental caries and periodontitis are primarily caused by indigenous oral bacteria. Generally, pathogenic bacteria often form three-dimensional structures (biofilms), consisting mainly of extracellular polysaccharides (EPS) that exhibit structural integrity and stability against antimicrobial agents and fungicides, thereby causing the infection. *Streptococcus mutans*, a pathogen implicated in dental caries and dental plaques, also forms robust biofilms on the tooth surface [1–3].

Antimicrobial-containing dentifrices are useful to prevent oral diseases involving biofilm formation. Dentifrices mainly include antimicrobials such as isopropyl methylphenol (IPMP), thymol, cetylpyridinium chloride, chlorhexidine gluconate, green tea extracts, and essential oils (such as monoterpenes) [4–6]. Notably, these antimicrobials inhibit biofilm formation by *S*. *mutans*, and some studies have investigated the underlying mechanism in the context of gene expression. For instance, as glucosyltransferase (GTF) synthesizes intracellular polysaccharides (IPS) and EPS, the suppression of biofilm-forming genes, such as *gtf*, could be important for biofilm inhibition. In addition, upregulation of the autolysin-like genes, such as N-acetylmuramoyl-L-alanine amidase type (*atlE*), superoxide dismutase (*sod*), and *ymc*A (general stress gene), increases cell death and general stress activity to suppress biofilm formation [7–9].

Among these antimicrobials, IPMP is widely used as an antimicrobial additive and preservative in dentifrices, disinfectants, and cosmetics because of its excellent antimicrobial effects against a wide variety of bacteria and fungi [4, 10, 11]. Further, IPMP is harmless to the human body, as it is a natural component of essential oils extracted from plants belonging to the Lamiaceae family (*Coridothymus capitatus* L.) [12–14]. In addition to antimicrobials, oral products also contain humectants, solubilizers, flavoring agents, preservatives, and surfactants. The role of IPMP in oral care products is particularly important in terms of sterilization and antibiofilm activity.

In this study, we investigated the inhibitory effects of IPMP and surfactant-containing IPMP-based agents on *S*. *mutans* biofilm formation in vitro. Notably, the combination of IPMP and a surfactant synergistically inhibited *S*. *mutans* biofilm formation. In addition, we also determined the genes involved in the mechanism underlying the inhibition of biofilm formation.

## Materials and methods

### Antimicrobial agents

IPMP powder (OSAKA KASEI CO., LTD., Osaka, Japan) and IPMP-based liquid agents (BIOSOL®-LIQUID, OSAKA KASEI) were used as antimicrobial agents, and their anti-biofilm effects were examined. The composition of the test samples is listed in Table 1.

**Table 1:** Composition of test samples used in this study.

Samples	Antimicrobials (AM)	Surfactant (Surf)	Solvent 1 (Sol 1)	Solvent 2 (Sol 2)	Composition (%)
					AM/Surf/Sol 1/Sol 2
A	IPMP	POEHCO^a^	IPG^b^	water	10/40/40/ 10
B	-	POEHCO	IPG	water	0/40/40/20
C	IPMP powder	-	-	-	100/ 0/0/0
D	-	POEHCO	-	water	0/10/0/90

^a^ Polyoxyethylene hydrogenated castor oil (NOF corporation, Tokyo, Japan)

Hydrophile Lipophile Balance (HLB): 14,0

^b^ Isoprene glycol (Kuraray Co., Ltd., Tokyo, Japan)

### Bacterial culture

The test bacterium *S*. *mutans* JCM 5705 was obtained from Biological Resource Center, National Institute of Technology and Evaluation, Tokyo, Japan. The *S*. *mutans* was grown in tryptic soy broth (TSB, Becton Dickinson, Franklin Lakes, NJ, USA) at 37°C in static conditions for pre-culture. For gene expression analysis and biofilm formation assays, bacteria were grown anaerobically in TSB and 0.25% sucrose-supplement TSB, respectively. The initial bacterial concentration was adjusted to approximately 10^6^ colony-forming units/mL.

### Quantitative evaluation of biofilm formation

The anti-biofilm effects of the antimicrobial agents against *S*. *mutans* were evaluated as follows: First, Sample A and B was diluted with sterile water (final IPMP concentration of sample A and B, 80 and 0 mg/mL, respectively), and the prepared sample solution was serially diluted two-fold with sterile water. Next, 2 μL of each dilution was plated into a 96-well plate (polystyrene), and 198 μL of *S*. *mutans* suspension was added to each well (final IPMP concentration of sample A, 800–1.6 μg/mL). For Sample C, 80 mg of IPMP powder was dissolved in 1 mL of dimethyl sulfoxide (DMSO, Fujifilm Wako Pure Chemical Co.). The prepared solution was serially diluted two-fold with sterile water and plated into a 96-well plate. The final concentration of IPMP in the culture medium was then adjusted to 800–1.6 μg/mL, the same as that in Sample A. For Samle D, 160 μL of 10% POEHCO solution was mixed with 40 μL of sterile water. The prepared solution was serially diluted two-fold with sterile water and plated into a 96-well plate. The 96-well plate was incubated at 37°C in an anaerobic atmosphere for 24 h. The measurement of biofilm amount using a crystal violet staining was performed based on H. M. Nassar *et al*. [15] with a slight modification. Thereafter, the culture broth was gently removed, and the wells were washed thrice with saline solution and stained with gentian violet solution (0.7% crystal violet in ethanol; Kishida Chemical, Osaka, Japan) for 20 min. Next, the staining solution was removed, and the wells were washed thrice with saline solution. The crystal violet retained by the biofilm was redissolved in 200 μL of ethanol. Finally, the absorbance was measured at 595 nm using a microplate reader (Nivo 3F, Perkin Elmer, Shelton, CT, USA). As the concentrations of DMSO and IPG used in this study were approximately 1%, neither solvent affected *S*. *mutans* biofilm formation.

In addition, two experimental procedures were carried out in this study to determine the surface modification effect of Sample A on *S*. *mutans* adhesion. In one procedure, 2 μL of Sample A was first added to a 96-well plate, as in the procedure described above, followed by the bacterial suspension. In the other procedure, the bacterial suspension was first added to the well plate, followed by the addition of sample A. Quantitative assessment of biofilm formation was performed as described above.

### Microscopic observation of *S*. *mutans* adhesion in the early-stage biofilm formation

The effect of surface modification by surfactants on the initial adhesion of *S*. *mutans* was investigated by microscopy. Three types of POECHO with hydrophilic-lipophilic balance (HLB) of 9.7, 14.0 and 16.5 were used as surfactants. The IPMP-based liquid agents used in this study contain PEOCHO with HLB: 14.0. Samples were prepared by the following two procedures. (1) the surfactant was dissolved in sterile water, and the surfactant solution was placed in a 96-well plate and then the bacterial suspension was added (process 1); (2) the bacterial suspension was first placed in a 96-well plate and then the surfactant solution was added (process 2). After 3 h incubation, the culture broth was removed and bacterial adherence was assessed using the crystal violet staining method. Bacterial adhesion to the well surfaces was then observed using a phase contrast microscope (CX43, Olympus, Tokyo, Japan).

### Quantitative real-time PCR measurements

*S*. *mutans* JCM 5705 was grown in TSB supplemented with a sub-minimum inhibitory concentration (sub-MIC) of IPMP (100 μg/mL). After 4 h of incubation, total RNA was immediately stabilized using the RNAprotect Bacteria Reagent (QIAGEN, Venlo, Netherlands). Cell pellets were resuspended in lysozyme TE buffer and Qiagen proteinase K and incubated at 23°C with gentle agitation for 10 min. The lysates were purified using an RNeasy Mini Kit (QIAGEN). Reverse transcription was performed using a PrimeScript II 1^st^ Strand cDNA Synthesis Kit (Takara Bio, Shiga, Japan) with reverse primers (1 μM each).

Real-time PCR was used to quantify *gtfB*, *gtfC*, and *gtfD* mRNA expression, with 16S rRNA used as an internal control. The primers targeting these genes were obtained from Sigma–Aldrich Corp. (St. Louis, MO, USA) (Table 2). Real-time PCR amplification was performed using the Mx3000P™ real-time PCR system (Agilent Technologies, Santa Clara, CA, USA). The reaction mixture (20 μL) contained Brilliant III Ultra-Fast QPCR/QRT-PCR Master Mix (Agilent Technologies), template cDNA, and forward and reverse primers (10 μM each). The PCR conditions involved an initial denaturation of 10 min at 95°C, followed by 45 cycles (denaturation: 95°C for 15 s and annealing and extension: 60°C for 1 min). An additional step, from 55°C for 30 s to 95°C for 30 s (0.2°C s^-1^), was performed to establish a melting curve. Threshold cycle values (*C*_*T*_) were determined, and the data were analyzed with the MxPro-Mx3000P Software v4.10 (Agilent Technologies) according to the 2^-ΔΔCT^ method.

**Table 2:** Nucleotide sequences of primers used in this study [13].

Gene	Description	Primer sequences (5′ → 3′)
16S rRNA	Normalizing internal standard	AGCGTTGTCCGGATTTATTG
		CTACGCATTTCACCGCTACA
*gtfB*	Glucosyltransferase B; glucan production	CACTATCGGCGGTTACGAAT
		CAATTTGGAGCAAGTCAGCA
*gtfC*	Glucosyltransferase C; glucan production	GATGCTGCAAACTTCGAACA
		TATTGACGCTGCGTTTCTTG
*gtfD*	Glucosyltransferase D; glucan production	TTGACGGTGTTCGTGTTGAT
		AAAGCGATAGGCGCAGTTTA

### Statistical analysis

The experiments were basically carried out more than thrice. For each experiment, data were summarized as the mean ± standard deviation. Student’s t-test was used to calculate the significance of the difference between the sample data and blank control data. The level of significance was set at a p value of < 0.05. P values that were considered significant for difference tests are provided in the figure caption.

## Results

### Quantitative evaluation for biofilm inhibition

**Fig 1:**
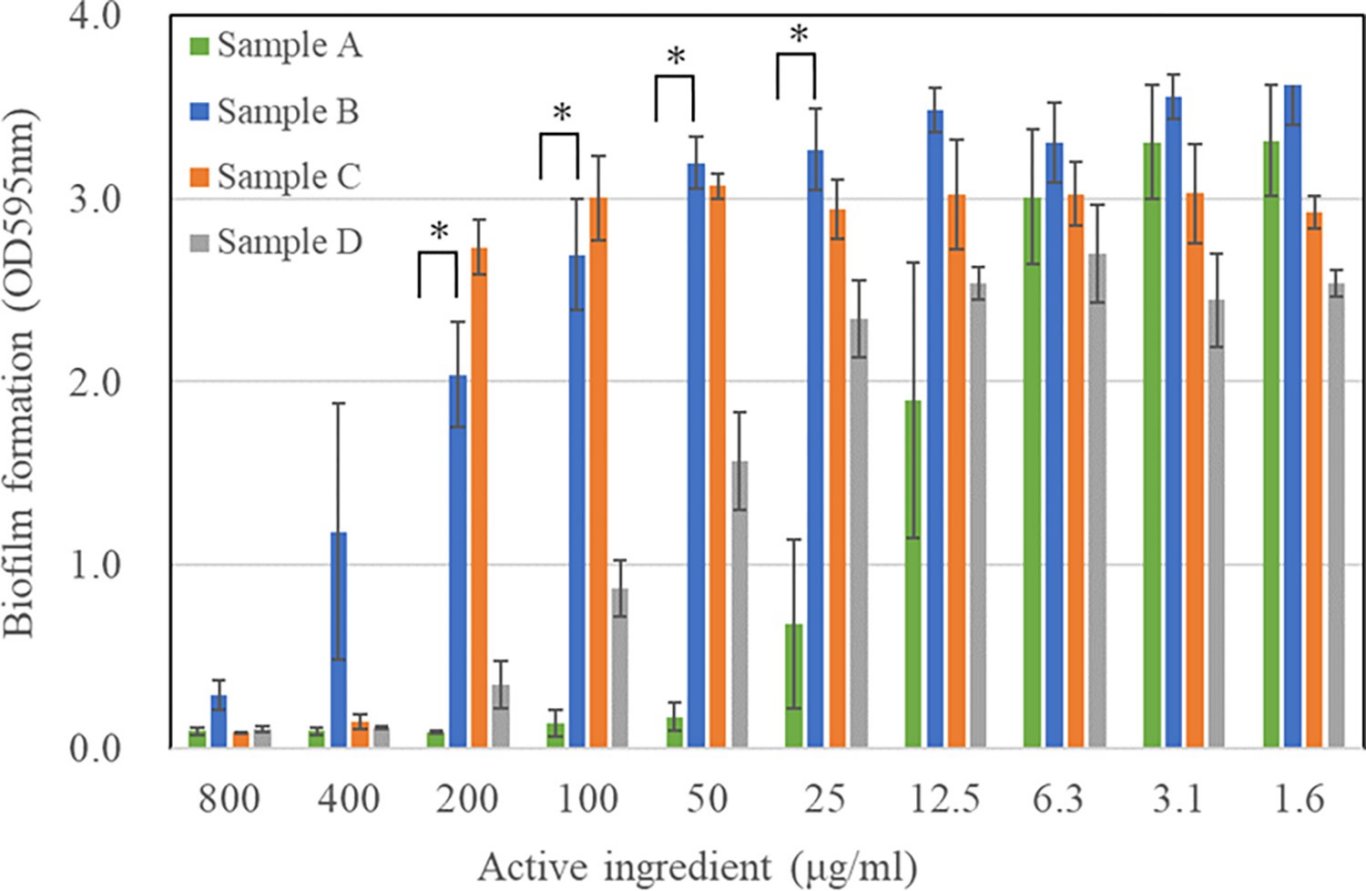
Inhibitory effects of IPMP-based agents on biofilm formation by *S*. *mutans*. The composition of the samples is as follows: Sample A (IPMP/ POEHCO/IPG), Sample B (-/POEHCO/IPG), Sample C (IPMP only), Sample D (POEHCO only). The horizontal axis indicates the concentration of IPMP in samples A and C. Although Sample B did not include IPMP, the other components in them were the same as in Sample A. In other words, as Sample B was the placebo of Sample A, the data of samples A and B were compared at the same horizontal axis for convenience. The experiment was done in triplicate and values are presented as mean ± S.D. error. Asterisk represents the p value < 0.05.

Crystal violet assay was performed to evaluate biofilm inhibition in the presence of the test antimicrobials. Fig 1 shows the different concentrations of active ingredients used to suppress biofilm growth. The composition of each test sample is listed in Table 1. The results revealed that, compared with Sample B (-/POEHCO/IPG), Sample A (IPMP/polyoxyethylene-hydrogenated castor oil [POEHCO]/IPG) was significantly more effective in inhibiting biofilm formation. In contrast, Sample C (IPMP only) exhibited a slight inhibitory effect in the sub-MIC concentration range (<100 μg/mL). Sample D (POEHCO only) exhibited a slight inhibitory effect in a high concentration range (>200 μg/mL) compared with Sample A. Here, we compare the effectiveness of the combination versus individual components in terms of the minimum biofilm inhibitory concentration (MBIC). In this case, the threshold absorbance value to determine the MBIC was set to 0.5. The ratio of MBIC (Sample C) / MBIC (Sample A) = 8, and the ratio of MBIC (Sample D) / MBIC (Sample A) = 4, which supports the effectiveness of the combination versus individual components. Incidentally, the biofilm formation with Sample A (50 μg/mL) was 5.5% compared to without samples. We speculate that the inhibition of biofilm formation in a high concentration is due to the prevention of bacterial attachment onto the surface modified by POEHCO (discussed below). These results indicate that the combination of IPMP and POEHCO exerted a synergistic effect.

Further, we calculated the fractional inhibitory concentration (FIC) index to evaluate the synergistic effects of IPMP-based agents [16] using the following formula:

$$FIC\;index=\frac{MIC\;of\;component\;A\;in\;combination\;}{MIC\;of\;component\;A\;only}+\frac{MIC\;of\;component\;B\;in\;combination}{MIC\;of\;component\;B\;only}$$

Based on the ΣFIC values, the interactions between components A and B were categorized into the following four effects: (1) Synergistic effect: When ΣFIC was ≤0.5, the effect of the two components in combination was much stronger than the sum of the effects of the single drug. (2) Additive effect: When ΣFIC was 0.5–1.0, two drugs in combination were slightly more effective than a single drug. (3) Indifferent effect: When ΣFIC was 1.0–2.0, the combined effect was neither improved nor inferior to that of the single agent. (4) Antagonistic effect: When ΣFIC was >2.0, the effect of the combination was inferior to the action of the single agent. In this study, the threshold absorbance value to determine the minimum biofilm inhibitory concentration was set to 0.5. The FIC index between IPMP and POEHCO was approximately 0.38, which supports a synergistic effect.

### Influence of surfactant on *S*. *mutans* attachment to well surfaces

**Fig 2:**
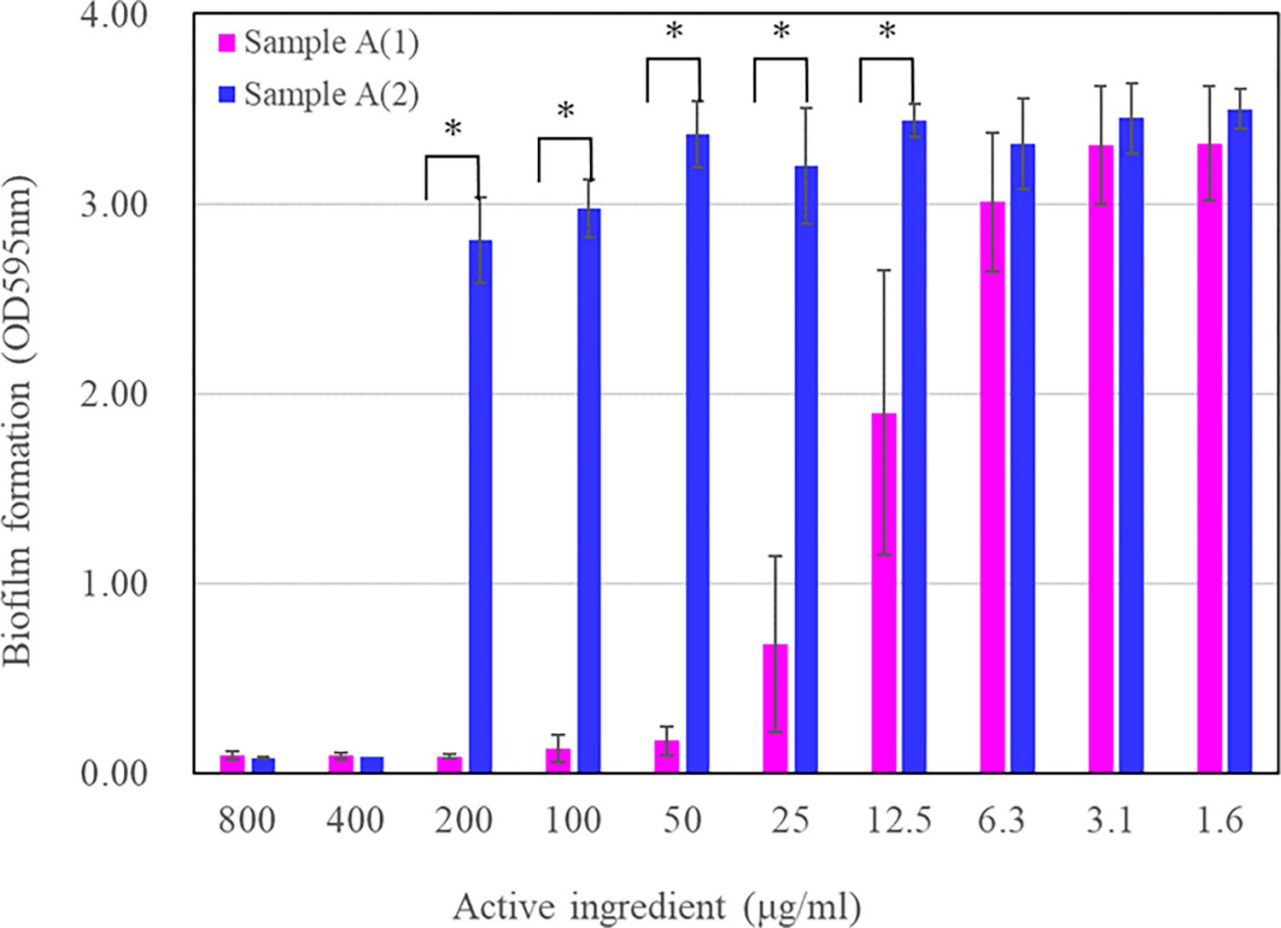
Inhibitory effect on biofilm formation by *S*. *mutans* by IPMP based agents. Sample A (1): Sample A (an IPMP-based agent dissolved in water) was added into a 96-well plate, followed by the addition of the culture solution. Sample A (2): The culture solution was added into a 96-well plate, followed by the addition of sample A (an IPMP-based agent). The experiment was done in triplicate and values are presented as mean ± S.D. error. Asterisk represents the p value < 0.05.

To clarify the surface modification effect of Sample A, the amount of biofilm formation was compared with or without treatment of the wells (Fig 2). The inhibition of Sample A (2) on biofilm formation was drastically reduced compared with that of Sample A (1). This suggests that Sample A may inhibit bacterial adhesion by masking the well surface as one mode of action.

**Fig 3:**
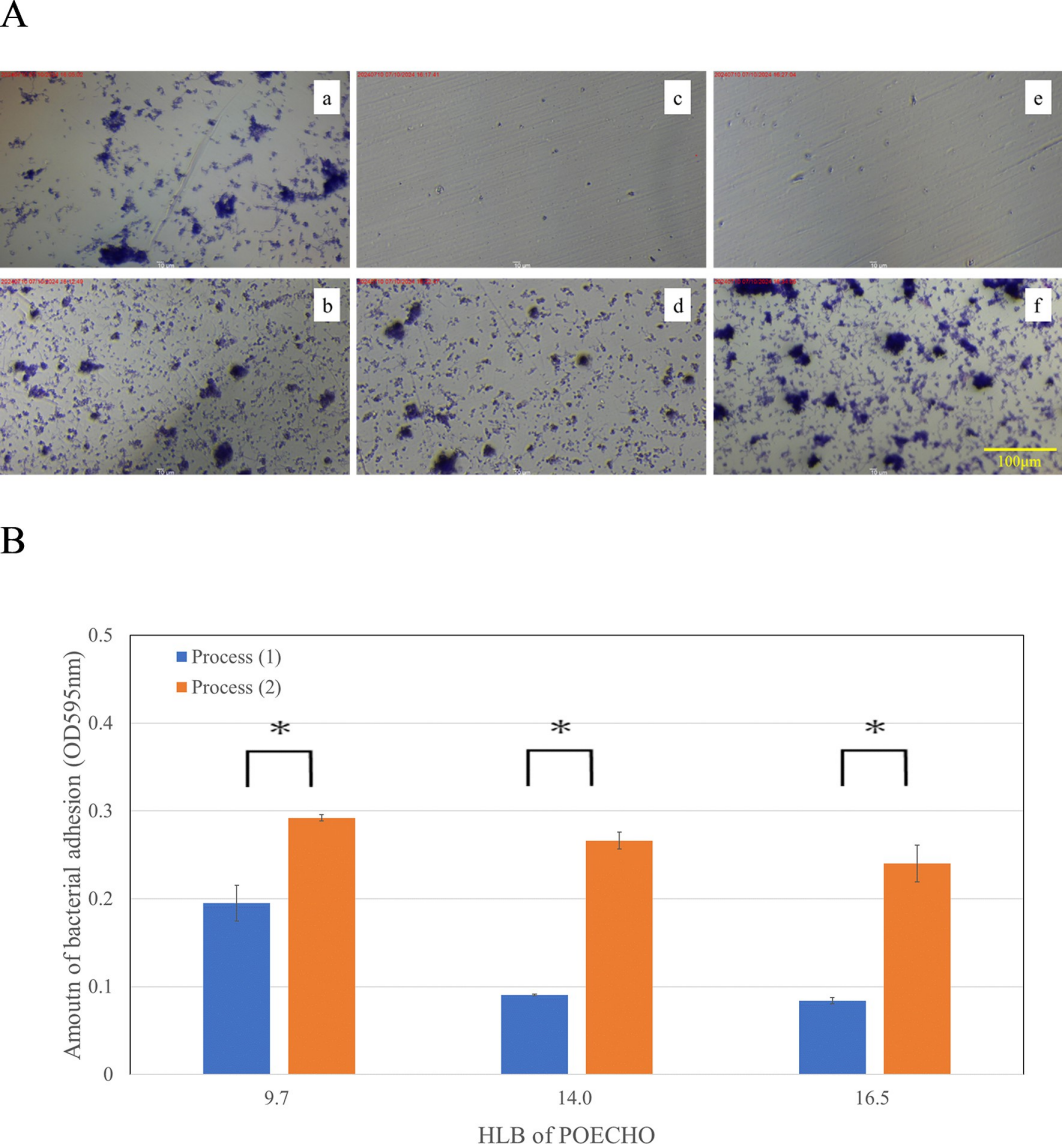
Microscopic observation of *S*. *mutans* attachment onto well surface (A), and quantitative analysis of the amount of *S*. *mutans* attachment onto well surface (B). **(A)** HLB 9.7: (a) Process (1), (b) Process (2), HLB 14.0: (c) Process (1), (d) Process (2), and HLB 16.5: (e) Process (1), (d) Process (2). Process (1): The surfactant solution was first placed in a 96-well plate, followed by the addition of the bacterial suspension. Process (2): The bacterial solution was placed in a 96-well plate, followed by the addition of the surfactant solution. The experiment was done in thrice and values are presented as mean ± S.D. error. Asterisk represents the p value < 0.05.

Next, the contribution of well masking by surfactants to the inhibition of bacterial adhesion was verified for POECHO showing three different HLBs (Fig 3). Crystal violet staining results showed that process (1) significantly inhibited bacterial adhesion to the well surface when compared to process (2) (Fig 3B). The more hydrophilic HLB: 14.0 and 16.5 POECHOs showed more effective inhibition than the less hydrophilic HLB: 9.7 POECHO. These trends were also confirmed by microscopic observations (Fig 3A). Pre-treatment of the surface with the more hydrophilic HLB: 14.0 and 16.5 POECHO resulted in clearly fewer bacterial flocs observed under other conditions.

### Influence of antimicrobial agents on gene expression

**Fig 4:**
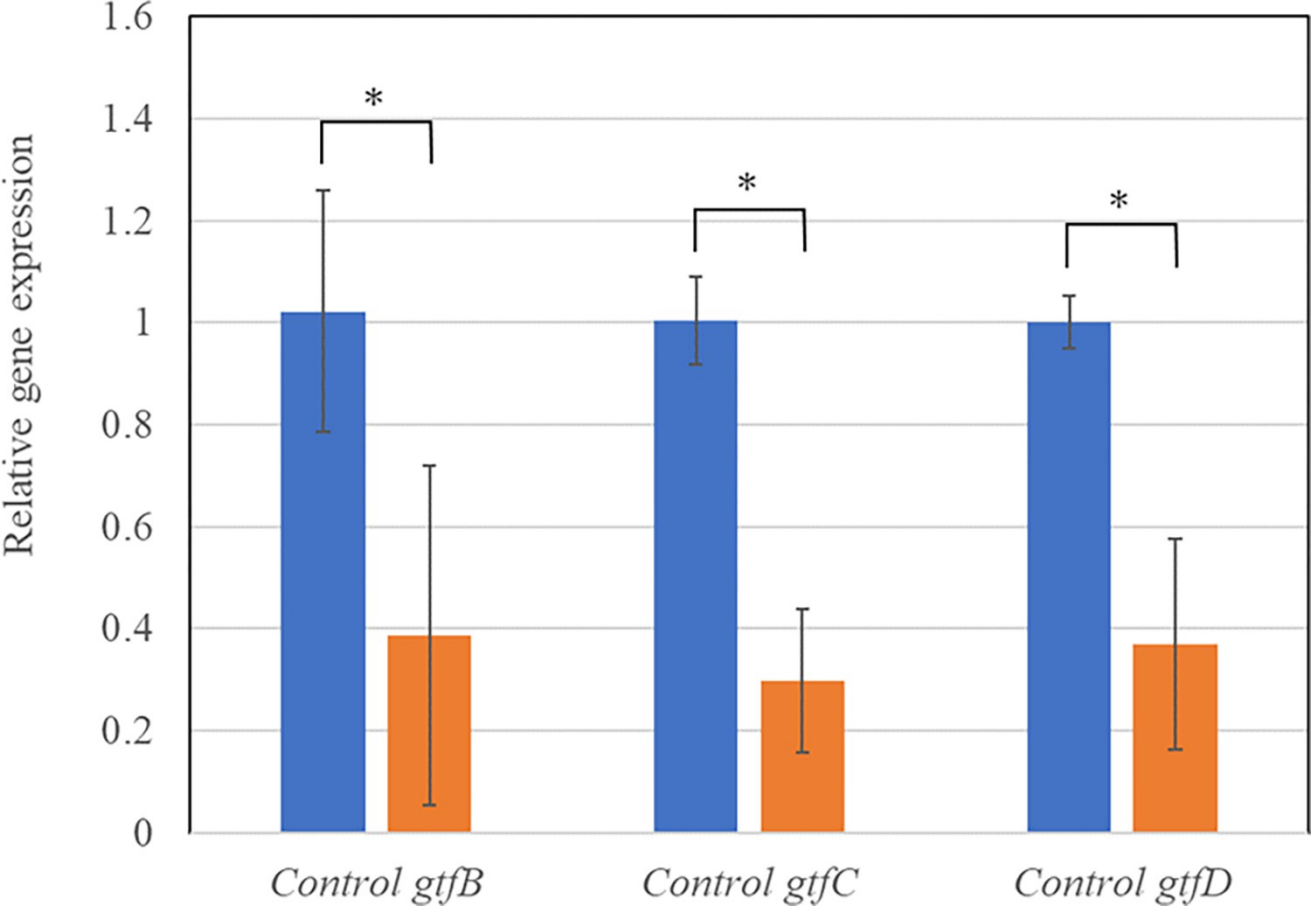
Influence of sub-MIC of IPMP on *gtfB*, *gtfC*, and *gtfD* gene expression in *S*. *mutans*. Significant suppression of gene expressions was observed compared with 16S rRNA as an internal control. The experiment was done in four times and values are presented as mean ± S.D. error. Asterisk represents the p value < 0.05.

To determine the inhibitory effect of IPMP on cell attachment and biofilm formation by *S*. *mutans*, real-time PCR was used to quantify *gtfB*, *gtfC*, and *gtfD* mRNA expression, with 16S rRNA as an internal control. Melting curves revealed the absence of non-specific products in all amplification reactions (data not shown). Compared to the untreated control, IPMP at sub-MIC levels (100 μg/L) significantly inhibited *gtfB*, *gtfC*, and *gtfD* gene expression by 62%, 70%, and 63%, respectively (Fig 4).

## Discussion

*S*. *mutans* is the most influential etiological bacteria in dental caries, which forms a robust biofilm on the tooth surface. Extracellular polymeric substances in biofilm protect the bacteria through a barrier function, thereby reducing the efficacy of antimicrobial agents in the treatment of infections. Consequently, efficient inhibition of biofilm formation is important to treat such infections. In this study, we investigated the inhibitory effects of IPMP-based agents consisting of IPMP and surfactants on *S*. *mutans* biofilm formation. The combination of IPMP and POEHCO showed a synergistic inhibitory effect on biofilm formation (FIC = 0.38), as evidenced by the significantly lower minimum biofilm inhibitory concentration in Sample A than in samples B, C and D (Fig 1). Interestingly, although POEHCO, a surfactant, did not exhibit antimicrobial activity against *S*. *mutans* in the concentration range investigated in the biofilm formation assay, it contributed to synergistic inhibition in this study.

Notably, we hypothesized a possible mechanism of synergistic biofilm inhibition by the combination of IPMP and POEHCO as follows. Generally, the initial process of biofilm formation involves the attachment of bacteria to the tooth surface, which is crucial for the subsequent formation of mature biofilms [17]. Allegrone *et al*. reported that coating silicone elastomer disks with certain biosurfactants inhibits *Staphylococcus aureus* adhesion and biofilm formation [18]. Further, biosurfactants alter the chemical and physical properties of tooth surface, such as roughness and hydrophobicity, to counteract microbial adhesion and biofilm formation [19, 20]. Moreover, IPMP exhibits antimicrobial activity against planktonic *S*. *mutans* [4]. In this study, bacterial adhesion may have been prevented by the altered properties of the well surface (polystyrene) because POECHCO was preferentially dispersed and adhered to the well surface. Notably, the surface modification by POECHCO and the antimicrobial effect of IPMP may have synergistically enhanced the anti-biofilm effect.

The influence of the order of agent addition on the biofilm inhibition is shown in Fig 2. This indicates that POEHCO may inhibit bacterial adhesion by masking the well surface and that dispersing POEHCO in the liquid leads to insufficient adherence of POEHCO on the well surface. Grivet *et al*. showed that *S*. *mutans* prefers hydrophobic surfaces [21, 22]. Further, HLB of POEHCO was approximately 14, indicating its hydrophilicity. Therefore, coating the well (polystyrene) surface with POEHCO may have changed the surface properties of the well to hydrophilic, thereby inhibiting *S*. *mutans* adhesion. To verify the above hypothesis, microscopic observation and quantitative comparison of *S*. *mutans* adhesion to well surface was carried out (Fig 3). In this experiment, three POECHOs exhibiting HLBs of 9.7, 14.0 and 16.5 were used. The results showed that when comparing process (1) with process (2), process (1) inhibited bacterial adhesion to the well surface. The effect was particularly high for HLB 14.0 and 16.5. This indicates that the more hydrophilic POECHO (Higher HLB) tended to inhibit bacterial adhesion to the well surface. These results support the above hypothesis. Limitations of this study was that the studies were only in vitro analyses with medium and micro-plate. Further studies are required to investigate the effect of POEHCO on the attachment of *S*. *mutans* to saliva-covered surfaces such as teeth, and clinical tests of synergistic *S*. *mutans* inhibition effect with combination of IPMP and POECO in oral care products.

During the early stages of *S*. *mutans* biofilm formation, the IPS and EPS produced by GTF are important for the formation of a three-dimensional firm matrix of *S*. *mutans* on the tooth surface. Therefore, in this study, we evaluated the effects of IPMP on the *gtf* genes of *S*. *mutans* using gene expression analysis. As shown in Fig 4, IPMP inhibited the expression of *gtfB*, *gtfC*, and *gtfD* at sub-MIC. A suppression of the *S*. *mutans gtfB* gene by the IPMP isomers thymol and carvacrol has also been reported previously [7]. Suppression of these genes inhibits polysaccharide synthesis, which may contribute to the synergistic effect of POEHCO on *S*. *mutans* surface attachment in this study. Nevertheless, further studies are required to validate our hypothesis that the adhesion of POEHCO to the well surface is critical for synergistic inhibition. However, the effect of POEHCO on the attachment of *S*. *mutans* to saliva-covered surfaces, such as teeth, needs further investigation.

## Conclusions

The combination of IPMP and POEHCO synergistically inhibited biofilm formation by *S*. *mutans*. This effect was synergistically enhanced by two factors: uniform adherence of POEHCO to the well surface may have inhibited bacterial attachment to the well surface and the antimicrobial activity of IPMP suppressed biofilm formation-related genes. Our findings indicate that IPMP-based agents are extremely useful dentifrices for the prevention of oral diseases involving biofilm formation.

## References

[1] Zayed SM, Aboulwafa MM, Hashem AM, Saleh SE. Bioflm formation by Streptococcus mutans and its inhibition by green tea extracts. AMB Express 2021; 11: 73. 10.1186/s13568-021-01232-6. 34032940 PMC8149520

[2] Inaba T, Obana N, Habe H, Nomura N. Biofilm formation by Streptococcus mutans is enhanced by indole via the quorum sensing pathway. Microbes Environ. 2020; 35(2). https://www.jstage.jst.go.jp/browse/jsme2. doi: 10.1264/jsme2.ME19164 32350164 PMC7308578

[3] Ahn S-J, Ahn S-J, Wen ZT., Brady L. J, Burne RA. Characteristics of biofilm formation by Streptococcus mutans in the presence of saliva. Infect Immun. 2008; 76(9): 4259–4268. doi: 10.1128/IAI.00422-08 18625741 PMC2519434

[4] Yoshikawa C, Hashimoto M, Iwasaki K, Kakimoto K. Antibacterial activity and biofilm dispersion of an isopropyl methylphenol-containing dentifrice. Nano Biomed. 2023; 15(1): 1–6.

[5] Fujiki M, Honda M. The investigation of synergistic activity of protamine with conventional antimicrobial agents against oral bacteria. Biochem Biophys Res Commun. 2020; 523: 561–566. doi: 10.1016/j.bbrc.2020.01.002 31932035

[6] Miladi H, Zmantar T, Kouidhi B, Al Qurashi YMA., Bakhrouf A, Chaabouni Y, et al. Synergistic effect of eugenol, carvacrol, thymol, p-cymene and g-terpinene on inhibition of drug resistance and biofilm formation of oral bacteria. Microb Pathog. 2017; 112: 156–163. 10.1016/j.micpath.2017.09.057. 28964825

[7] Khan ST, Khan M, Ahmad J, Wahab R, Abd‑Elkader OH., Musarrat J, et al. Thymol and carvacrol induce autolysis, stress, growth inhibition and reduce the biofilm formation by Streptococcus mutans. AMB Expr. 2017: 7:49 doi: 10.1186/s13568-017-0344-y 28233286 PMC5323333

[8] Adil M, Singh K, Verma PK., Khan AU. Eugenol-induced suppression of biofilm-forming genes in Streptococcus mutans: An approach to inhibit biofilms. J Glob Antimicrob Resist. 2014; 2: 286–292. doi: 10.1016/j.jgar.2014.05.006 27873689

[9] Xu X, Zhou X D., Wu C D. Tea catechin epigallocatechin gallate inhibits Streptococcus mutans biofilm formation by suppressing gtf genes. Arch oral biol. 2012; 57: 678–683. doi: 10.1016/j.archoralbio.2011.10.021 22169220

[10] Park Y-D, Cho J-W, Seo W-S, Park H-H, Shin S-C, Sano H, et al. Clinical study on effects of dentifrice containing 0.05% isopropyl methylphenol (IPMP) and 0.05% dipotassium glycyrrhizinate (GK2) on gingival conditions. International J Clinic Prev Dentist. 2010;6(2):55–61.

[11] Ohara H, Odanaka K, Shiine M, Hayasaka M, Antimicrobial effect of oral care gel containing hinokitiol and 4-isopropyl-3-methylphenol against intraoral pathogenic microorganisms. PLOS ONE 18(9): e0283295. doi: 10.1371/journal.pone.0283295 37656688 PMC10473516

[12] Juan RA, Olga PA, Mirian PP. Chemical composition and anti-trypanosoma cruzi effect of thymus vulgaris L. (thyme) essential oil and its main component, thymol, in mice. Amer J Pharm Pharmac. 2015; 2(4): 21–27.

[13] Mohamed A, Maroua Y1, Olfa B, Bilel C, Manef A. GC-MS identification and in vitro evaluation of antioxidant activity of bioactive molecules extracted from Mentha pulegium L. and Thymus capitatus native to Tunisia. The Pharm Chem J. 2018; 5(5):1–8.

[14] Alexa E, Sumalan RM, Danciu C, Obistioiu D, Negrea M, Poiana M-A, et al. Synergistic antifungal, allelopatic and anti-proliferative potential of salvia officinalis L., and thymus vulgaris L. essential oils. Molecules 2018; 23: 185, doi: 10.3390/molecules23010185 29337923 PMC6017077

[15] Nassar Hani M., Li Mingyun, Gregory Richard L. Effect of Honey on Streptococcus mutans Growth and Biofilm Formation. Appl. Environ. Microbiol. 2012; 78(2): 536–540. doi: 10.1128/AEM.05538-11 22038612 PMC3255743

[16] Okukawa M, Yoshizaki Y, Yano S, Nonomura Y. The selective antibacterial activity of the mixed systems containing myristic acid against staphylococci. J Oleo Sci. doi: 10.5650/jos.ess21090 34373405

[17] Burne RA., Ahn S-J, Wen ZT., Zeng L, Lemos JA., Abranches J, et al. Opportunities for disrupting cariogenic biofilms. Adv Dent Res. 2009; 21(1): 17–20. doi: 10.1177/0895937409335593 19710079 PMC2853230

[18] Allegrone G, Ceresa C, Rinaldi M, Fracchia L. Diverse effects of natural and synthetic surfactants on the inhibition of staphylococcus aureus biofilm. Pharmaceutics 2021; 13: 1172. doi: 10.3390/pharmaceutics13081172 34452132 PMC8402037

[19] Rodrigues L.R., Banat I.M., van der Mei H.C., Teixeira J.A., Oliveira R. Interference in adhesion of bacteria and yeasts isolated from explanted voice prostheses to silicone rubber by rhamnolipid biosurfactants Get access Arrow. J Appl Microbiol. 2006; 100(3): 470–80. doi: 10.1111/j.1365-2672.2005.02826.x 16478486

[20] Elshikh M, Marchant R, Banat IM. Biosurfactants: promising bioactive molecules for oral-related health applications. FEMS Microbiol Lett. 2016;363(18):fnw213. doi: 10.1093/femsle/fnw213 Epub 2016 Sep 11. 27619892

[21] Grivet M., Morrier J. J., Benay G., Barsotti O. Effect of hydrophobicity on in vitro streptococcal adhesion to dental alloys. J mater sci mater med. 2000;11: 637–642. doi: 10.1023/a:1008913915399 15348088

[22] Westergren G., Olsson J. Hydrophobicity and adherence of oral streptococci after repeated subculture in vitro. Infect Immun. 1983; 40(1): 432–435. doi: 10.1128/iai.40.1.432-435.1983 6832836 PMC264867

